# EMG1 is essential for mouse pre-implantation embryo development

**DOI:** 10.1186/1471-213X-10-99

**Published:** 2010-09-21

**Authors:** Xiaoli Wu, Sumit Sandhu, Nehal Patel, Barbara Triggs-Raine, Hao Ding

**Affiliations:** 1 Department of Biochemistry and Medical Genetics, University of Manitoba, 745 Bannatyne Avenue, Winnipeg R3E 0J9, Canada; 2 Department of Pediatrics & Child Health, University of Manitoba, 840 Sherbrook Street, Winnipeg R3A 1S1, Canada; 3 Center for the Investigation of Genetic Disease, Manitoba Institute of Child Health, 715 McDermot Avenue, Winnipeg R3E 3P4, Canada

## Abstract

**Background:**

Essential for mitotic growth 1 (EMG1) is a highly conserved nucleolar protein identified in yeast to have a critical function in ribosome biogenesis. A mutation in the human EMG1 homolog causes Bowen-Conradi syndrome (BCS), a developmental disorder characterized by severe growth failure and psychomotor retardation leading to death in early childhood. To begin to understand the role of EMG1 in mammalian development, and how its deficiency could lead to Bowen-Conradi syndrome, we have used mouse as a model. The expression of *Emg1 *during mouse development was examined and mice carrying a null mutation for *Emg1 *were generated and characterized.

**Results:**

Our studies indicated that *Emg1 *is broadly expressed during early mouse embryonic development. However, in late embryonic stages and during postnatal development, *Emg1 *exhibited specific expression patterns. To assess a developmental role for EMG1 *in vivo*, we exploited a mouse gene-targeting approach. Loss of EMG1 function in mice arrested embryonic development prior to the blastocyst stage. The arrested *Emg1^-/- ^*embryos exhibited defects in early cell lineage-specification as well as in nucleologenesis. Further, loss of p53, which has been shown to rescue some phenotypes resulting from defects in ribosome biogenesis, failed to rescue the *Emg1^-/- ^*pre-implantation lethality.

**Conclusion:**

Our data demonstrate that *Emg1 *is highly expressed during mouse embryonic development, and essential for mouse pre-implantation development. The absolute requirement for EMG1 in early embryonic development is consistent with its essential role in yeast. Further, our findings also lend support to the previous study that showed Bowen-Conradi syndrome results from a partial EMG1 deficiency. A complete deficiency would not be expected to be compatible with a live birth.

## Background

Ribosome biogenesis is fundamental to cell growth and accounts for a substantial proportion of a cell's energy expenditure [1]. The ribosomal RNAs (rRNAs) are central to the ribosome structure and function [2]. The rRNA genes exist as tandem repeats and form the foci upon which the nucleoli form. The rRNA precursor (47S) is synthesized from the genes by RNA polymerase I and assembled with ribosomal proteins to form the 90S pre-ribosome. This 90S preribosome is matured to form the large-60S ribosomal subunit and the small-40S ribosomal subunit. The 60S subunit contains the 28S, 5.8S and 5S rRNAs as well as approximately 49 proteins, whereas the 40S subunit contains the 18S rRNA and approximately 33 proteins. It is estimated that 200 proteins are involved in assembling the mature ribosomes [3]. Many of them have been studied in yeast, but not in mammals. Nonetheless, the proteins are highly conserved and as a starting point, it is reasonable to assume that they function similarly in mammals.

EMG1 (also known as Nep1) was initially identified as "Essential for Mitotic Growth" in yeast [4], and later was shown to be involved in the biogenesis of the mature 40S ribosome [5,6]. Yeast EMG1 (yEMG1) is a 28 kDa protein primarily detected in the nucleolus [5,6]. Because the deletion of y*EMG1 *in yeast is lethal, temperature sensitive mutations in this gene have been used to study the effects of its deficiency. Depletion of yEMG1 resulted in a reduction in 18S rRNA, a decrease in 40S ribosomal subunits and an increase in the ratio of 60S to 40S ribosomal subunits [5,6]. These findings indicate an important role for EMG1 in the biogenesis of the 40S ribosome.

Deciphering the precise role of EMG1 in 40S ribosome biogenesis has been challenging. A temperature sensitive mutation in yEMG1 could be suppressed by the methyl donor S-adenosyl methionine (SAM) [6] or deletion of the snR57 gene encoding a snoRNA needed for 2'-O-ribose-methylation of G1570 in the 18S rRNA [7]. Furthermore, yEMG1 was found to interact directly with snoRNA [8] and the 18S rRNA [9]. Taken together, these findings suggested that yEMG1 functions to methylate the 18S rRNA, a concept that was later supported by the identification of yEMG1 as a SAM-dependent pseudouridine-N1-specific methyltransferase [10].

The EMG1 protein is highly conserved from archaebacteria to humans or mice [11]. Expression of the human orthologue of EMG1 in yeast demonstrates that it is capable of suppressing the lethal defect in *yEMG1 *cells, indicating that EMG1 is both structurally and functionally conserved among these eukaryotes [6]. More recently, a mutation in human *EMG1*, which significantly reduces EMG1 protein levels, has been found to cause Bowen-Conradi syndrome (BCS), an autosomal recessive disorder characterized by severely impaired prenatal and postnatal growth, profound psychomotor retardation, and death in early childhood [12]. This finding strongly suggests that EMG1, as a key molecule in ribosomal synthesis, could be important for development. To get a better understanding of this, in the present study, we have attempted to generate an EMG1-deficient mouse and characterize the expression of *Emg1 *during mouse development. Our data demonstrates that EMG1 is essential for mouse pre-implantation development.

## Results and Discussion

### Expression of *Emg1 *during mouse embryogenesis

**Figure 1:**
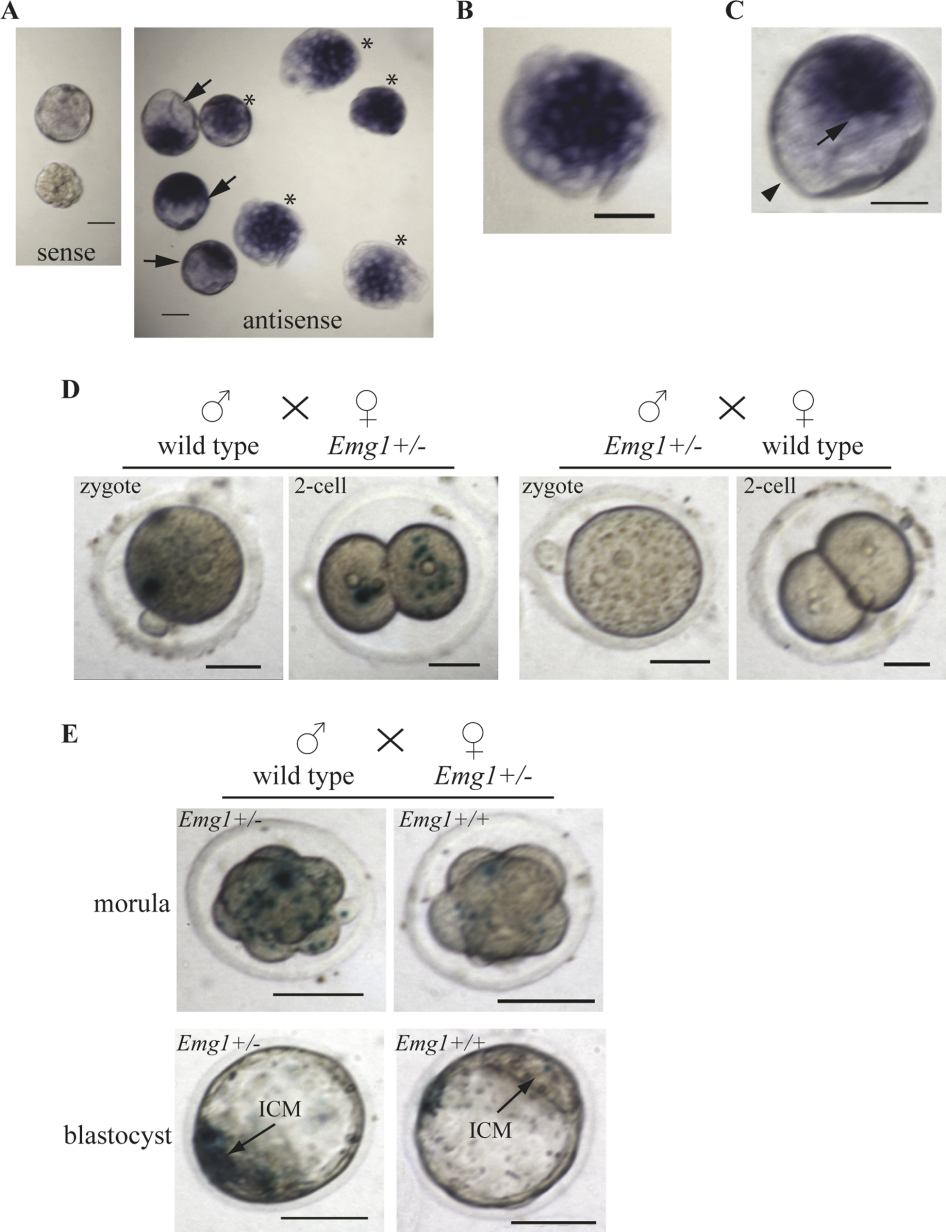
**Expression of *Emg1 *in pre-implantation embryos**. (A) Whole mount RNA *in situ *hybridization of mouse pre-implantation embryos. Asterisks indicate the E2.5 morulae, and arrows indicate the blastocysts. Hybridization with *Emg1 *sense probe is presented as a control. (B) High magnification showing that a stronger *Emg1 *signal is detected within the inside cells at the morula stage. (C) High magnification demonstrating that the inner cell mass of the mouse blastocyst is strongly stained with *Emg1 *antisense probe (arrow indicates), whereas the trophectoderm has a very low level of *Emg1 *(arrowhead). (D) Whole mount X-gal staining of zygotes and 2-cell embryos either from the cross of wild-type males with *Emg1^+/- ^*females or from *Emg1^+/- ^*males with wild-type females. Only the embryos from the breeding of wild-type males with *Emg1^+/- ^*females show LacZ signals. (E) X-gal staining of morulae and blastocysts collected from the breeding of wild-type males with *Emg1^+/- ^*females, shows wide-spread LacZ signals in the 8-cell morula and in the ICM of *Emg1^+/-^*. In *Emg1^+/+ ^*morulae or blastocysts, only a few cells are weakly stained for LacZ, probably reflecting a low level of residual maternally transmitted LacZ protein in these cells. Scale bar in A-D, 50μm, and in E, 100μm.

**Figure 2:**
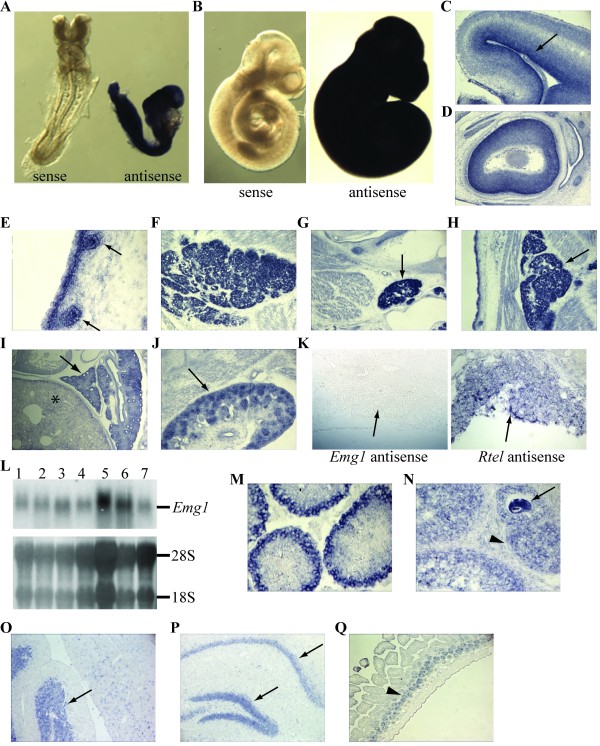
**Expression of *Emg1 *in post-implantation embryos and adult mice**. (A-B) Whole mount RNA *in situ *hybridization of post-implantation mouse embryos (A: E8.5; B: E9.5). (C-J) RNA *in situ *hybridization on the sections of E13.5-E15.5 mouse embryos, demonstrating that *Emg1 *is expressed at low levels in most tissues, but strongly in the ventricular zone of the neuroepithelium (C, arrow indicates), the neural layer of retina (D), the follicles of vibrissae (E, arrows indicate), thymus (F), submandibular glands (G, arrow indicates), brown adipose tissue (H, arrow indicates), lung (I, arrow indicates. Asterisk indicates liver in which low expression of *Emg1 *was found), nephric tubules and renal mesenchyme (J, arrow indicates). (K) RNA *in situ *hybridization on the sections of E9.5 placenta, showing that *Emg1 *mRNA is low or undetectable in the trophoblast cells (arrow indicates). *Rtel *antisense probe is presented as a positive control. Magnification for images C-K: x10. (L) Northern blot analysis of *Emg1 *expression in mouse adult tissues. Lane: 1 Brain; 2 Lung; 3 Heart; 4 Liver; 5 Spleen; 6 Kidney; 7 Skeletal muscles. Lower panel shows the total ribosomal RNAs for normalization. (M) RNA *in situ *hybridization on mouse testis, showing that *Emg1 *is highly and specifically expressed in spermatogonia and meiotic spermatocytes. (N) In ovary, strong *Emg1 *expression is detected in oocyte (arrow indicates). The granulose cells of the pre-antral follicles are also positive for *Emg1 *(arrowheads). (O-P) In brain, *Emg1 *is predominately expressed in the granular neurons of cerebellum (O, arrow indicates) and the neurons located in hippocampus (P, arrows indicate). (Q) In intestine, *Emg1 *expression was mainly identified in the crypts (arrowhead). Magnification for images O-Q: x10.

As a first step toward elucidating the role of EMG1 during development, we analyzed the expression of the *Emg1 *gene in mouse embryos and postnatal tissues. Using RNA *in situ *hybridization, *Emg1 *expression was readily detected in E2.5 morula embryos (Figure 1A), with the strongest expression associated with the inside cells in the late stage of morula (Figure 1B). High levels of *Emg1 *mRNA were also detected in the inner cell mass (ICM) of mouse blastocysts (E3.5), but not in the trophectoderm (Figure 1C). This expression pattern is consistent with *Emg1 *being expressed highly in the embryo proper, but weakly expressed or not detectable in the trophoblasts of mouse placenta (Figure 2).

**Figure 3:**
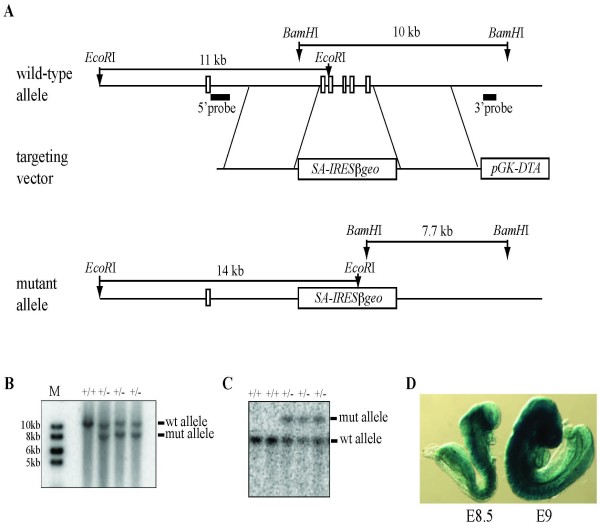
**Targeted disruption of EMG1 in mice by homologous recombination**. (A) Structure of the wild-type *Emg1 *locus, the targeting vector and the mutated locus with exons 2-6 replaced by *SA-IRESβgeo *cassette. The location of the hybridization probes (5' probe and 3' probe) for Southern blot analyses are shown. (B-C) Southern blot analysis of targeted ES cell clones using the 3' probe (B) and the 5' probe (C). (D) Whole mount X-gal staining of *Emg1^+/- ^*embryos (E8.5 and E9).

Expression of *Emg1 *in mouse pre-implantation embryos was also analyzed by determining the activity of LacZ in *Emg1 *knockout heterozygotes in which the expression of the *LacZ *transgene was regulated by the endogenous *Emg1 *regulatory elements (see Figure 3). Crosses of wild-type males with *Emg1^+/- ^*females revealed a positive LacZ signal in zygotes and in 2-cell embryos, which was not present in those from breeding *Emg1^+/- ^*males with wild-type females (Figure 1D), indicating that these early mouse embryos could contain maternally transmitted EMG1. However, starting at the 8-cell morula stage, there were two distinct LacZ-staining patterns for the embryos collected from the breeding of wild-type males with *Emg1^+/- ^*females (Figure 1E). Among these embryos, *Emg1^+/- ^*showed wide-spread LacZ staining, whereas the ones that were genotyped as *Emg1^+/+ ^*exhibited a weak disperse staining which could be from residual maternally transmitted LacZ proteins. This data indicates that the onset of zygotic expression of EMG1 may occur at the 8-cell morula stage. In addition, X-gal staining of E3.5 *Emg1^+/- ^*blastocysts showed strong LacZ staining in the ICM, but not in the trophectoderm (Figure 1E), which is consistent with *Emg1 *expression detected by *in situ *hybridization (Figure 1C).

To determine the expression pattern of *Emg1 *in post-implantation embryos, mouse embryos at E8.5-E15.5 were analyzed. At E8.5-E9.5, *Emg1 *was widely and strongly expressed and showed no clear tissue-specific pattern (Figure 2A-B). At later stages (E11.5-E15.5), however, *Emg1 *was found to be expressed at a low level in most embryonic tissues, but strongly in several regions, including the ventricular zone of the neuroepithelium (Figure 2C), the neural layer of retina (Figure 2D), the follicles of vibrissae (Figure 2E), thymus (Figure 2F), submandibular glands (Figure 2G), brown adipose tissue (Figure 2H), lung (Figure 2I), nephric tubules and renal mesenchyme (Figure 2J) and seminiferous tubules in the testis (not shown).

To examine the expression of *Emg1 *in extraembryonic tissue, we performed RNA *in situ *hybridization on E8.5-E9.5 mouse placenta. No *Emg1 *signal was detected in the trophoblast cells, while a control gene, *Rtel *(*R*egulator of *Te*lomere *l*ength) showed strong expression in this cell lineage (Figure 2K) [13].

In adult mice, similar levels of *Emg1 *mRNA were detected in multiple tissues by Northern blot hybridization (Figure 2L), suggesting that EMG1 could be widely expressed during postnatal development. However, using RNA *in situ *hybridization assays, *Emg1 *cell-specific expression patterns were detected in several tissues (Figure 2M-Q). In the adult testis, *Emg1 *is highly and specifically expressed in both spermatogonia and early meiotic spermatocytes, but not in late stage spermatocytes (Figure 2M). A strong *Emg1 *signal was also identified in oocytes and the granulosa cells of the pre-antral follicles in the ovary (Figure 2N). In the adult mouse brain, *Emg1 *expression was mainly detected in the granular layer of neurons of the cerebellum (Figure 2O) as well as in the hippocampus (Figure 2P). Specific expression of *Emg1 *was also found in the crypts of the intestine (Figure 2Q).

Taken together, our gene expression data indicates that EMG1 is broadly distributed in the early developing embryos, but its expression is more restricted in the later stages of development. In adult mice, *Emg1 *also exhibits cell-specific expression, most notably in the gonads, brain and intestine. This expression pattern suggests that EMG1 may not only be important for early embryonic development, but could also be required for the development of several cell lineages at late developmental stages or during postnatal development.

### Generation of the *Emg1 *null mouse allele

To further study the developmental role of EMG1, we have mutated *Emg1 *in mice by homologous recombination. The mouse *Emg1 *gene contains 6 exons that encode a protein composed of 244 amino acids. In order to create an *Emg1 *null allele, a gene-targeting vector with a splice acceptor (SA)-*IRES*-*βgeo*-*pA *cassette was used to replace exons 2-6 and to remove approximately 80% of the *Emg1 *coding sequence (Figure 3A). Given that *Emg1 *is highly expressed in ES cells, the inserted *SA-IRESβgeo *cassette in the first intron of the *Emg1 *locus will trap exon 1 to turn on the expression of βgeo, a fusion protein of LacZ and neo. This was designed to allow us to significantly increase the targeting frequency, while also allowing us to establish a mouse allele in which a LacZ reporter is regulated by the endogenous *Emg1 *regulatory elements. Indeed, approximately 25% (11 out of 45) of the ES colonies obtained after G418 selection showed correct homologous recombination by Southern blot analysis using both 5' and 3' probes external to the targeting vector (Figure 3B-C). Furthermore, LacZ transgene expression in *Emg^+/- ^*embryos was entirely consistent with the expression pattern established by *in situ *hybridization (Figure 1E and 3D). In this study, we have used two independently targeted ES lines to generate germline-transmitting chimeras that were bred with 129S1 or CD1 females to produce *Emg1 *null mutants for functional analysis.

### The pre-implantation lethality of *Emg1 *null mutants

Both *Emg1^+/- ^*males and females were normal and fertile, and transmitted the targeted allele to about 50% of their progeny. However, no homozygous offspring were born from *Emg1^+/- ^*intercrosses (Table 1, n = 237), indicating that loss of EMG1 function leads to embryonic lethality. To determine at which stage of development the homozygous mutant embryos died, timed heterozygous matings were performed. An analysis of embryos between E8.5-E12.5 revealed that none of the 64 embryos genotyped were *Emg1^-/-^*. The expected Mendelian frequency of heterozygote, homozygote and wild-type was only observed in the pre-implantation embryos (Table 1), indicating that mice lacking EMG1 protein stopped developing at the pre- and/or peri-implantation stages.

**Table 1:** Genotype analysis of progeny from *Emg^+/- ^*intercrosses

Developmental Stage	No. of mice by genotyping			Total
	+/+	+/-	-/-	
Postnatal	107	130	0	237
E12.5	6	10	0	16
E9.5	4	13	0	17
E8.5	11	20	0	31
E3.5	11	22	9	42
E2.5	12	17	11	40

**Figure 4:**
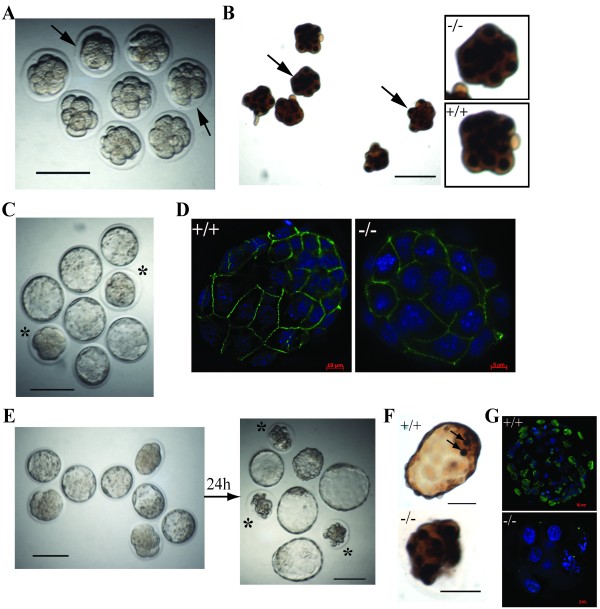
**Loss of EMG1 function results in pre-implantation arrest**. (A) E2.5 embryos collected from *Emg1^+/- ^*intercross. These embryos are indistinguishable from each other, despite the presence *Emg1^-/- ^*mutations among these progeny (labeled by arrows). (B) E2.5 *Emg^-/- ^*embryos (indicated by arrows) display the similar BrdU incorporation as *Emg1^+/+ ^*or *Emg1^+/- ^*littermates. (C) E3.5 embryos collected from *Emg1^+/- ^*intercross, showing that *Emg1^-/- ^*embryos arrest at the morula stage (labeled by asterisks), and fail to form an inner cell mass, trophectoderm cell layer and blastocoel cavity as wild-type or *Emg1^+/- ^*embryos. (D) Immunofluoresence with anti-E-cadherin antibody, showing that *Emg^-/- ^*morulae exhibit the same organized pattern of E-cadherin along the cell boundaries as wild-type littermates. (E) *In vitro *culture of E3.5 embryos from *Emg1^+/- ^*intercross. None of *Emg1^-/- ^*embryos develop into blastocyst (indicated by asterisks). (F) Whole mount TUNEL assay shows the high levels of apoptosis in E3.5 *Emg1^-/- ^*embryos after 24-hour culture. Few TUNEL-positive cells (indicated by arrows) are detected in wild-type littermate. (G) BrdU labeling assay to demonstrate the lack of cell proliferation in the cultured mutant embryos. Green: BrdU; Blue: Dapi. Scale bar in A-C and E-F, 100μm.

To address this further, we analyzed more than 100 pre-implantation embryos collected from two independent mouse lines carrying the *Emg1 *knockout allele. At E2.5, *Emg1^+/- ^*intercrosses yielded early stage embryos that were indistinguishable from each other, despite the presence of *Emg1^-/- ^*mutations among these progeny (Figure 4A). In addition, all the *Emg1^-/- ^*embryos at this developmental stage showed similar BrdU incorporation (Figure 4B), and nearly undetectable levels of apoptosis, like that of the wild-type controls (data not shown), indicating that *Emg1^-/- ^*embryos were not blocked at the E2.5 stage.

The E3.5 embryos from *Emg1^+/- ^*intercrosses, however, consistently showed a mixture of morula and blastocyst-stage embryos. Genotyping of these embryos demonstrated that whereas the blastocysts were either *Emg1^+/+ ^*or *Emg1^+/- ^*heterozygotes, the morula-stage embryos were *Emg1^-/- ^*mutants (Figure 4C). In *Emg1^-/- ^*morulae, the blastomeres flattened and tightly aligned themselves against each other to form a compact ball of cells. Immunofluoresence (IF) with anti-E-cadherin antibody revealed that *Emg^-/- ^*morulae exhibited the same organized pattern of E-cadherin along the cell boundaries as the wild-type control (Figure 4D). These data indicate that *Emg^-/- ^*embryos do reach the compaction stage of morula development.

To determine whether *Emg1^-/- ^*embryos at E3.5 were arrested at this stage or simply delayed, we further cultured these embryos *in vitro*. After 24 h of culture, while *Emg1^+/+ ^*and *Emg1^+/- ^*E3.5 embryos formed expanded blastocysts, none of the E3.5 *Emg1^-/- ^*embryos developed to blastocysts (Figure 4E). Instead, many cells in the cultured mutant embryos showed fragmented, pyknotic nuclei, suggestive of cell death. To determine whether cell death is indeed increased in these cultured mutant embryos, we performed a TUNEL assay. A significantly higher number of TUNEL-positive nuclei were detected in the mutant embryos than in *Emg1^+/+ ^*embryos (Figure 4F). In addition, very few cells in the cultured mutant embryos were positive in the BrdU labeling assay (Figure 4G). These data clearly indicate that *Emg1^-/- ^*embryos arrest prior to forming the blastocyst and subsequently the embryos undergo degeneration. Because of this severe phenotype, no *Emg1*-deficient cells could be established to perform further molecular investigations.

### Specification of early cell lineages in *Emg1^-/- ^*embryos

**Figure 5:**
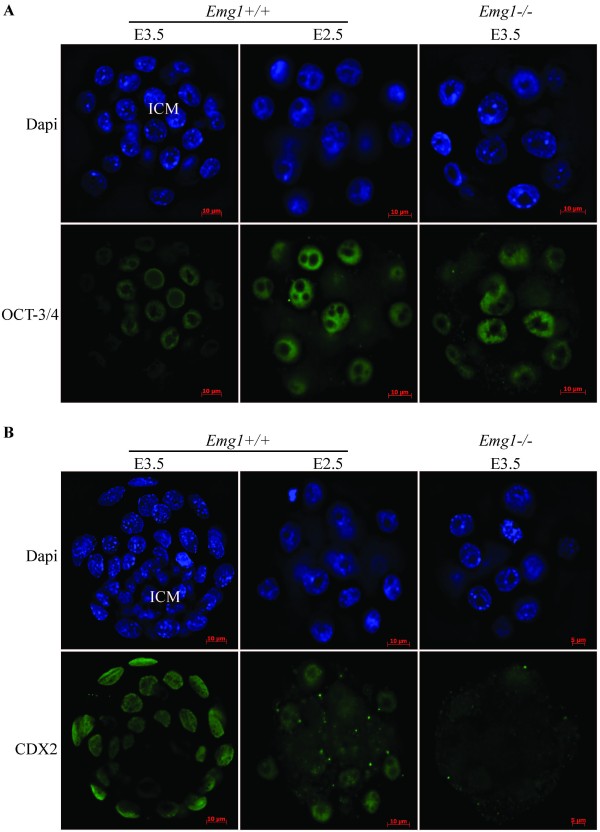
**Characterization of the early embryonic lineages in *Emg1^-/- ^*mutant embryos**. (A) Immunofluorescence staining with anti-OCT-3/4 antibody, demonstrating that E3.5 *Emg1^-/- ^*embryos do not form the OCT-3/4-positive inner cell mass (ICM) like the wild type blastocyst, but display a similar level of OCT-3/4 expression as wild type controls. (B) Immunofluorescence staining with anti-CDX2 antibody, showing that CDX2 is weakly expressed in the blastomeres of mutant embryos as compared to *Emg1^+/+ ^*blastocysts or morulae.

The failure of *Emg1^-/- ^*embryos to form blastocysts could suggest a defect in the specification of early cell lineages in these mutant embryos. To test this, we analyzed the expression of several early cell lineage markers, including OCT4, NANOG and CDX2, in E3.5 *Emg1^-/- ^*embryos. These markers have been shown to be widely expressed in the blastomeres of cleavage stage embryos, but become restricted to the different lineages, i.e. OCT4 and NANOG in ICM and CDX2 in trophectoderm, after initiation of blastocyst formation [14-17]. Using these lineage markers, we found that although E3.5 *Emg1^-/- ^*embryos did not form the OCT-3/4 or NANOG-positive ICM like that of *Emg1^+/+ ^*blastocysts, the blastomeres in the mutant embryos displayed levels of expression of these markers similar to wild type (Figure 5A and Additional file 1). However, the expression of CDX2 was consistently found to be significantly decreased in the mutant embryos as compared to the controls (Figure 5B). These results suggest that loss of EMG1 function more specifically down-regulates the expression of CDX2 in the blastomeres of the cleavage stage embryos, which might then influence the allocation or morphogenesis of cell lineages during early embryogenesis.

### Ribosomal biogenesis in *Emg1^-/- ^*mutants

EMG1 has been shown to be a highly conserved nucleolar protein required for ribosome biogenesis [5]. *Emg1-*null mutants exhibit arrested development prior to the blastocyst stage, similar to that observed in other mouse models that lack factors involved in ribosomal RNA synthesis or processing, including RBM19 (RNA-binding motif protein 19) [18], pescadillo-1 (PES-1) [19], fibrillarin [20], RNA polymerase I or II [21], BYSL [22], SURF6 [23] and RPS19 (ribosomal protein S19) [24]. Some of these genetic mutations have been clearly demonstrated to cause severe defects in ribosomal biogenesis [19,21,22]. Thus, loss of EMG1 function in mice could also disrupt this biological pathway, leading to pre-implantation lethality.

**Figure 6:**
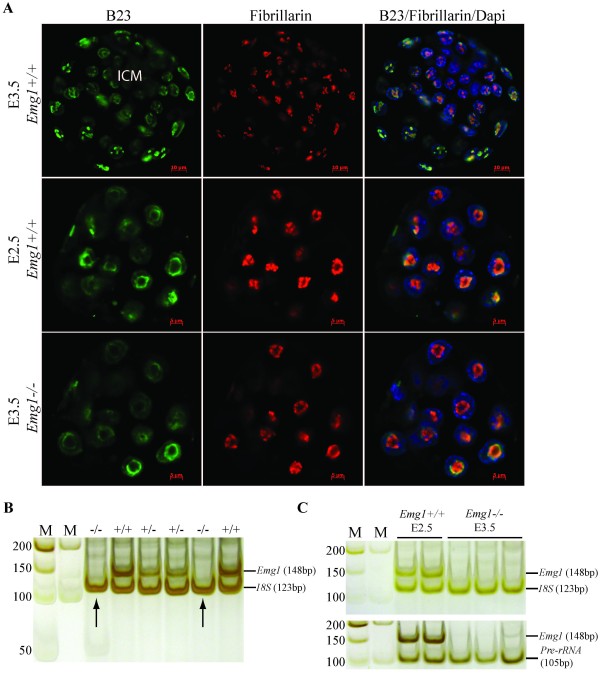
**Characterization of nucleologenesis and ribosomal synthesis in *Emg1^-/- ^*embryos**. (A) Immunofluorescence staining for the nucleolar markers B23/nucleophosmin and fibrillarin. In E3.5 *Emg1^+/+ ^*embryos, the nucleoli are smaller and more irregular than the NPBs in E2.5 morulae. The nucleoli in E3.5 *Emg1^-/- ^*mutants, however, display a large sphere shape, which closely resemble NPBs as seen in E2.5 wild-type embryos. (B) RT-PCR on E2.5 morulae harvested from *Emg1^+/- ^*intercrosses. Arrows indicate the morulae that lack *Emg1 *mRNA but show a similar level of 18S rRNA to that of *Emg1*-expressing morulae. The genotypes for each sample are also presented. M: 50 bp or 100 bp DNA markers. (C) RT-PCR on E3.5 *Emg^-/- ^*embryos, showing similar levels of 18S rRNA and pre-rRNA in both mutant and wild-type embryos.

To address this question, we first determined whether loss of EMG1 could affect nucleologenesis during pre-implantation development. As demonstrated previously, "de novo" nucleologenesis which begins at the two-cell stage is critical for the resumption of rRNA transcription during early embryogenesis [25-27]. This process involves the morphological transformation of the nucleolus precursor body (NPB) to a mature, tripartite nucleolus as seen in the blastocysts [27,28]. To determine the effect of the *Emg1 *gene-deletion on nucleologenesis, we performed IF staining for the nucleolar makers B23/nucleophosmin and fibrillarin in E3.5 embryos harvested from *Emg1^+/- ^*intercrosses. As shown in Figure 6, in E3.5 *Emg1^+/+ ^*or *Emg1^+/- ^*embryos, the nucleoli are smaller and more irregular than NPBs in E2.5 morula, indicating a striking maturation occurred at this developmental stage. In contrast, the nucleoli in E3.5 *Emg1^-/- ^*embryos still showed the large ring shape, closely resembling the NPBs observed in E2.5 wild-type morula. Therefore, these data strongly suggest that deletion of the *Emg1 *gene arrests nucleologenesis during early embryonic development.

To determine if *Emg1^-/-^*embryos exhibit defective 40S ribosome biogenesis, similar to yeast depleted in yEMG1, we examined the level of mature 18S rRNA using reverse transcription (RT) followed by PCR. Although the levels of 18S rRNA are significantly reduced in yeast depleted in yEMG1, no detectable decrease in 18S rRNA were detected in *Emg1^-/- ^*morulae at E2.5 as compared to wild-type embryos at the same developmental stage (Figure 6B). Since E2.5 mouse morulae could contain residual maternal rRNAs, we used the same approach to analyze 18S rRNA in E3.5 *Emg1^-/- ^*embryos as compared to wild-type embryos with the same developmental stage. Again, no difference was observed (Figure 6C). Given that the levels of 18S rRNA in cells are very high, and small differences would not be detectable using this assay, we also looked for an increase in the precursors to the 18S rRNA, the pre-rRNA, but again no obvious difference between *Emg1^-/- ^*and wild type or heterozygous embryos was detected (Figure 6C). The unchanged expression of 18S rRNA and 47S rRNA in *Emg1^-/- ^*embryos was also indicated by RT followed by real-time PCR analysis (data not shown). Although this data differs from that in yeast, it is still possible that there is a delay in ribosomal RNA processing or assembly that was not detected using these assays. More sensitive approaches such as metabolic labeling or pre-rRNA specific probes may be required to show a delay in rRNA processing, as was recently shown in the study of a protein required for the maturation of the 60S ribosomal subunit in human cells [29]. However, due to the early pre-implantation lethality of the *Emg1 *null allele, we are unable to derive EMG1-deficient cells in which to perform these assays. Future experiments with a conditional knockout of EMG1 will greatly help to address the role of EMG1 in the regulation of ribosomal biogenesis during development.

### P53 deficiency does not rescue the pre-implantation arrest of *Emg1^-/- ^*mice

Previous studies have found that mutations in many proteins involved in ribosome biogenesis lead to an up-regulation of p53 [30-32], a key regulator of the cell cycle and apoptosis. The importance of p53 in the regulation of ribosome biogenesis has been addressed by studies showing that inhibition of p53 can suppress the effects of some defects in ribosome biogenesis. In mice, p53 inhibition was found to suppress the effects of mutation of *Tcof *(Treacher Collins syndrome-causing gene) [33] and *Rps24 *(Diamond-Blackfan anemia-causing gene) [34]. These findings suggest that the inhibition of p53 may suppress the detrimental effects of mutations in other disorders of ribosomal biogenesis, such as EMG1 deficiency. To test this, E3.5 embryos were collected from intercrosses of *Emg1/p53 *double heterozygotes (*Emg1^+/-^/p53^+/-^*) or *Emg1^+/-^/p53^-/-^*. A total of 5 *Emg1^-/-^/p53^-/- ^*E3.5 embryos were identified, and all of them were found to be arrested at morula stage like the *Emg^-/- ^*null mutants (Additional file 2). In addition, none of E3.5 *Emg1^-/-^/p53^-/- ^*embryos developed to blastocysts during *in vitro *culture. Taken together, these data demonstrate that p53 inactivation fails to rescue the pre-implantation arrest of the *Emg1 *null allele.

In summary, we have demonstrated that EMG1 is essential for mouse pre-implantation. We showed that loss of EMG1 function specifically arrests early embryonic development at the morula stage, preventing blastocyst formation. This phenotype is consistent with our expression data showing that *Emg1 *is highly expressed during this critical developmental stage. However, due to the high expression of *Emg1 *in mouse oocytes (Figure 2N), which could be maternally transmitted into early developing embryos like other nucleolar components [35] (Figure 1D), we could not exclude the possibility that EMG1 is also required before the morula stage. Future experiments with a conditional knockout of EMG1 specifically in mouse oocytes will allow us to answer this question. Nevertheless, our study highlights a critical role of EMG1 in mouse early embryonic development.

The importance of EMG1 in development has also been demonstrated by our recent finding that this gene is mutated in human BCS syndrome, a severe developmental disorder with prenatal and postnatal growth retardation, profound psychomotor deficit, and death in early childhood [36]. Because of this mutation, the EMG1 protein was found to be significantly reduced in fibroblasts of BCS patients [12]. The residual protein that is detected is likely necessary to allow survival, as mice with a complete deficiency of EMG1 exhibit pre-implantation lethality. Therefore, the involvement of EMG1 in development could be dose-dependant. The hypomorphic mutation of EMG1 in BCS could specifically affect late embryonic development or certain cell lineages to cause BCS-associated phenotypes. In line with this, in this study, we found that *Emg1 *is predominately expressed in distinct cell types at late embryonic developmental stages or in adult (Figure 2). The unique expression of *Emg1 *in the granular neurons of cerebellum or in hippocampus could underly an important role for EMG1 in the control of psychomotor development, whose dysfunction is characteristic of BCS. Future experiments with mouse alleles to allow knockin of the BCS mutation, or a conditional allele, will allow us to address the pathological role(s) of EMG1 *in vivo*.

## Conclusions

We have provided direct genetic evidence that EMG1 is essential for mouse pre-implantation. Given the requirement of yEMG1 in the biogenesis of the ribosomal 40S subunit, our study also highlights the critical role of ribosomal biogenesis in early development. The absolute requirement for EMG1 in mouse development is consistent with its essential role in yeast. Further, our findings also lend support to the previous study that showed Bowen-Conradi syndrome results from a partial EMG1 deficiency. A complete deficiency would not be expected to be compatible with a live birth.

## Methods

### Construction of the *Emg1 *gene-targeting vector

The *Emg1 *gene-targeting vector was made based on a PCR-based cloning strategy as described previously [37]. Briefly, the mouse *Emg1 *genomic fragments required for the 5' and 3' arms of homology were PCR-amplified from the genomic DNA of R1 ES cells (on 129S1 background) with a high-fidelity polymerase (Clontech). After validation by DNA sequencing, the PCR products were cloned into two individual vectors that contain the *SA-IRESβgeo *cassette, and a *pGKDTA *fragment (the negative selection cassette), respectively. Subsequently, using the restriction enzymes and the cloning strategy as described [37], the DNA fragments were isolated, and assembled together to generate the gene-targeting vector.

### Generation of *Emg1 *deficient mice

The *Emg1 *gene-targeting construct was linearized and electroporated into R1 ES cells, and then selected with G418 (250 μg/ml). The G418 resistant ES clones were screened by Southern blot analysis for the correctly targeted allele using *Eco*R1 (for the 5' external probe) and *Bam*H1 (for the 3' external probe) digestion. Two independently targeted ES cell clones were used to generate chimeric mice that subsequently transmitted the genetic alternation through the germ line. The phenotypes of *Emg1^-/- ^*mutants derived from both targeted ES cell lines were indistinguishable. Mice were maintained on either 129S1 or on a mixed 129S1 and CD1/ICR background, in which *Emg1^-/- ^*developed the same phenotype. All mouse experiments were performed in accordance with procedures approved by the University of Manitoba Animal Care and Use Committee.

### Genotyping

PCR and Southern blot analysis were applied for genotyping the *Emg1 *heterozygous mice. PCR was performed on ear-punched DNA. Primers to amplify the targeted allele were the sense primer (P1), located in intron 2 (5'-GTTCCTCAGCATATACGTGCT-3') and antisense primer (P2) specific for the *SA-IRESβgeopA *cassette (5'-GGGACAGGATAAGTATGACATCA-3'). To detect the wild-type *Emg1 *allele, P1 primer and an antisense primer (P3) locating in exon 2 (5'-TGTAAACCTGTAGCAAGCCAGCT-3') were used for PCR. Southern blot analysis was undertaken using standard protocols.

To genotype pre-implantation mouse embryos, a nested PCR method was applied. DNA was prepared by incubating E2.5-E3.5 embryos with 10 μl of Proteinase K buffer (10 mM Tris pH 8.3, 50 mM KCl, 2 mM MgCl_2_, 0.01% gelatin, 0.45% Nonidet P40, 0.45% Tween 20 and 500 μg/ml proteinase K) for 1 h at 55°C followed by incubation at 95°C for 10 min. 5 μl of DNA sample was then directly used for PCR using P1 and P2 (for the *Emg1 *targeted allele) and P1 and P3 (for the wild-type allele). 2 μl of products from the first round PCR were further PCR-amplified with the internal primers, generating 264 bp (wild-type allele) and 353 bp (targeted allele) products, respectively.

A similar approach was also applied to genotype the *p53 *null allele in *Emg1/p53 *double mutant pre-implantation embryos. The first round PCR was done using the primers as described previously [38]. In the second round PCR, the following internal primers were utilized: 5'-TACCTCACTACAGGTGACCTG-3' (sense) and 5'-TCTTAGAGACAGTTGACTCCAG-3' (antisense) (for detecting the p53 wild-type allele), and 5'- TACCTCACTACAGGTGACCTG-3' (sense) and 5'-GTGATATTGCTGAAGAGCTTGG-3' (antisense) (for detecting the p53 null allele).

### Early embryo isolation and *in vitro *culture

*Emg1^+/- ^*or *Emg1^+/-^/p53^+/- ^*mice were intercrossed, and the females were examined for the presence of a vaginal plug which was set as embryonic day (0.5). Embryos at different stages of development (E2.5 through E3.5) were collected by either dissecting ampullae or flushing oviducts with M2 medium (Millipore). For *in vitro *culturing, E3.5 embryos were placed in KSOM-1/2AA medium (Millipore), and incubated at 37°C for 24 h.

### Immuno-staining of pre-implantation embryos

Immuno-staining of pre-implantation embryos was performed based on the protocols provided by Dr. Janet Rossant http://www.sickkids.ca/research/rossant/custom/protocols.asp. The following antibodies were used: monoclonal anti-CDX2 (1:200, CDX2-88, BioGenex, CA, USA), monoclonal mouse anti-OCT3/4 (1:50, C10; Santa Cruz Biotechnology), rabbit anti-NANOG (1:200, Cosmo Bio), Rat anti-E-cadherin (1:100, Sigma), monoclonal mouse anti-B23 (1:50, Invitrogen) and rabbit anti-fibrillarin (1:100, Abcam). Secondary antibodies included Texas Red or Alexa488-conjugated goat anti-mouse, goat anti-rat and goat anti-rabbit (Molecular Probes). To visualize nuclei, embryos were stained with DAPI (0.5μg/ml) for 3 min at room temperature. Immuno-stained embryos were mounted onto microscopic slides with ProLong Gold (Invitrogen) and covered with glass cover slips. Images were collected using a Zeiss Axioplan 2 microscope to generate Z-stacks which were deconvolved using iterative algorithms program in Axio Vision 4.6.

For the BrdU labeling assay, E2.5-E3.5 embryos were incubated with KSOM-1/2AA medium containing 10 μM BrdU (Sigma) for 3 h, and were then fixed in 4% paraformaldehyde (PFA) for 10 min, permeabilized in 0.25% Triton X-100 for 10 min, treated with 2N HCl for 10 min, and detected with anti-BrdU antibody (Sigma).

### TUNEL staining

E2.5-E3.5 mouse embryos were fixed in 4% PFA in PBS for 1 h at room temperature, and permeabilized for 1 h in PBS-0.5% Triton X-100. The embryos were then washed three times in PBS-0.1% Trion X-100 and incubated at 37°C for 1 h in a staining solution containing biotin-dUTP, terminal deoxynucleotide transferase (TdT), and detected using an ABC staining kit (Vector).

### Whole mount x-gal staining

Pre-implantation embryos were fixed for 2 min with 1% PFA, 0.2% glutaraldehyde and 0.02% NP40 in PBS. After fixation, embryos were washed three times with PBS containing 0.02% NP40, and stained at 37°C overnight with a staining solution (4 mM K_4_Fe(CN)_6_, 4 mM K_3_Fe(CN)_6_, 2 mM MgCl_2_, and 0.2% X-gal in PBS). For E8.5-E9.5 embryos, embryos were fixed for 30 min with 4% PFA. After extensive washing with PBS containing 0.02% NP40 (three times, 20 min/each time), embryos were stained as described above.

### RNA *in situ *hybridization

RNA *in situ *analysis of whole mount mouse embryos and frozen sections of mouse tissues were performed according to established protocols [39] with antisense and sense digoxigenin-labeled riboprobes which were *in vitro *transcribed from the full-length mouse *Emg1 *coding sequence. Mouse embryos were collected from pregnant outbred ICR female mice at E9.5-E15.5 days of gestation, and the adult tissues were harvested from two-month old ICR mice. All the samples were fixed in DEPC-treated 4% PFA at 4°C overnight.

RNA *in situ *hybridization on early mouse embryos (E2.5-E3.5) was performed essentially based on a described protocol [40]. To preserve the pre-implantation embryos, the whole procedure was carried out in a transwell-insert (Corning).

### Northern Blot Analysis

Total RNA from flash-frozen mouse tissues was extracted using TRIzol (Life Technologies, Inc.). 20 μg of total RNA was separated on a 1% agarose-formaldehyde gel and transferred to Hybond nylon membrane (Amersham). Hybridization was carried out in PerfectHyb (Sigma) with 1.5×10^6 ^cpm/ml probe which covers the whole coding sequence of *Emg1*.

### RT-PCR analysis of pre-implantation embryos

Each blastocyst or morula was lysed with 5 μl ice-cold Cell Lysis II Buffer (Ambion) for 10 min at 75°C. 2 μl of lysate was used for PCR based genotyping, the rest was digested with DNase1 (0.08 unit/μl, Ambion) at 37°C for 15 min. After inactivation at 75°C for 5 min, 2 μl of DNase1-treated embryonic lysate was used in an RT reaction in a 10 μl volume using the OneStep RT-PCR kit (Qiagen) according to the manufacturer's instructions. The final concentration of specific primers (see the reverse primers described below) was 0.6 μM each. 2 μl RT mixture were then used for PCR reactions with the Multiplex PCR kit (Qiagen) and the primers described below. The PCR cycles contain an initial denaturation step of 95°C for 15 min and 40 cycles of 94°C for 30 s, 60°C for 90 s, and 72°C for 60 s, and a final 10 min extension step at 72°C. The PCR products were separated on a 12% polyacrylamide gel in 1× Tris-borate-EDTA buffer. The bands on the gel were visualized by the silver staining method as described [41].

The following primers were used for the above RT-PCR analysis: Mouse *Emg1*: forward (5'-TGAAGTGAACCCCCAGACTC-3') and reverse (5'-GAAGTGGTCGGACACTGGAT-3'). The amplified DNA band is 148 bp. Mouse pre-rRNA: forward (5'-CTCCTGTCTGTGGTGTCCAA-3') and reverse (5'-TGATACGGGCAGACACAGAA-3') in the 5' external transcribed spacer (5'-ETS) region of mouse 47S pre-rRNA [42]. The size of the PCR product is 105 bp. Mouse 18S rRNA: forward (5'-GCAATTATTCCCCATGAACG-3') and reverse (5'-GGCCTCACTAAACCATCCAA-3'), which gives rise to a DNA band with 123 bp.

## Abbreviations

EMG1: Essential for mitotic growth 1; BCS: Bowen-Conradi syndrome; rRNAs: ribosomal RNAs; SAM: S-adenosyl methionine; ICM: inner cell mass; PES-1: pescadillo-1; Rbm19: RNA-binding motif protein 19; Rps19: ribosomal protein S19; IF: immunofluorescence; PFA: paraformaldehyde; TdT: Terminal deoxynucleotide transferase; RT: reverse transcription.

## Authors' contributions

XW, BT and HD conceived and designed the experiments. XW and SS performed *in situ *and Northern hybridization. XW, SS and HD generated the gene-targeting vector and produced the knockout mice. XW and NP performed mouse breeding and genotyping and XW did all the analysis of the pre-implantation mouse embryos. BT and HD wrote the manuscript with subsequent contributions from all authors. All authors read and approved the final manuscript.

## Supplementary Material

Additional file 1 **Expression of NANOG in *Emg1^-/- ^*mutant embryos**. E3.5 embryos from *Emg1^+/- ^*intercross were co-immunostained with anti-NANOG (red) and anti-β-catenin (green) antibodies. In E3.5 *Emg1^+/+ ^*blastocysts, nuclear-localized NANOG is mainly found in the ICM. NANOG is also detected in the blastomeres of E2.5 *Emg1^+/+ ^*morulae. At E3.5, *Emg1^-/- ^*embryos arrest at the morula stage, in which the blastomeres express similar levels of NANOG as that in E2.5 *Emg1^+/+ ^*morulae. Click here for file

Additional file 2 **p53 inactivation fails to rescue the pre-implantation arrest of the *Emg1 *null allele**. E3.5 embryos were collected from intercross of *Emg1^+/-^/p53^+/- ^*(A) and cross of *Emg1^+/-^/p53^-/-^*(male) with *Emg1^+/-^/p53^+/- ^*(female) (B). In both, *Emg1^-/-^/p53^-/- ^*embryos show the same morula arrest as *Emg^-/-^/p53^+/- ^*or *Emg^-/-^*embryos. Scale bar, 100μm. Click here for file

## References

[B1] Warner JR The economics of ribosome biosynthesis in yeast Trends Biochem Sci 1999 24 437 440 10.1016/S0968-0004(99)01460-7 10542411

[B2] Moss T Langlois F Gagnon-Kugler T Stefanovsky V A housekeeper with power of attorney: the rRNA genes in ribosome biogenesis Cell Mol Life Sci 2007 64 29 49 10.1007/s00018-006-6278-1 17171232 PMC11136161

[B3] Connolly K Culver G Deconstructing ribosome construction Trends Biochem Sci 2009 34 256 263 10.1016/j.tibs.2009.01.011 19376708 PMC3711711

[B4] Hakuno F Hughes DA Yamamoto M The Schizosaccharomyces pombe mra1 gene, which is required for cell growth and mating, can suppress the mating inefficiency caused by a deficit in the Ras1 activity Genes Cells 1996 1 303 315 10.1046/j.1365-2443.1996.27029.x 9133664

[B5] Liu PC Thiele DJ Novel stress-responsive genes EMG1 and NOP14 encode conserved, interacting proteins required for 40S ribosome biogenesis Mol Biol Cell 2001 12 3644 3657 11694595 10.1091/mbc.12.11.3644 PMC60282

[B6] Eschrich D Buchhaupt M Kotter P Entian KD Nep1p (Emg1p), a novel protein conserved in eukaryotes and archaea, is involved in ribosome biogenesis Curr Genet 2002 40 326 338 10.1007/s00294-001-0269-4 11935223

[B7] Lowe TM Eddy SR A computational screen for methylation guide snoRNAs in yeast Science 1999 283 1168 1171 10.1126/science.283.5405.1168 10024243

[B8] Bernstein KA Gallagher JE Mitchell BM Granneman S Baserga SJ The small-subunit processome is a ribosome assembly intermediate Eukaryot Cell 2004 3 1619 1626 10.1128/EC.3.6.1619-1626.2004 15590835 PMC539036

[B9] Buchhaupt M Meyer B Kotter P Entian KD Genetic evidence for 18S rRNA binding and an Rps19p assembly function of yeast nucleolar protein Nep1p Mol Genet Genomics 2006 276 273 284 10.1007/s00438-006-0132-x 16721597

[B10] Wurm JP Meyer B Bahr U Held M Frolow O Kotter P Engels JW Heckel A Karas M Entian KD Wohnert J The ribosome assembly factor Nep1 responsible for Bowen-Conradi syndrome is a pseudouridine-N1-specific methyltransferase Nucleic Acids Res 2010 20047967 10.1093/nar/gkp1189 PMC2853112

[B11] Taylor AB Meyer B Leal BZ Kotter P Schirf V Demeler B Hart PJ Entian KD Wohnert J The crystal structure of Nep1 reveals an extended SPOUT-class methyltransferase fold and a pre-organized SAM-binding site Nucleic Acids Res 2008 36 1542 1554 10.1093/nar/gkm1172 18208838 PMC2275143

[B12] Armistead J Khatkar S Meyer B Mark BL Patel N Coghlan G Lamont RE Liu S Wiechert J Cattini PA Koetter P Wrogemann K Greenberg CR Entian KD Zelinski T Triggs-Raine B Mutation of a gene essential for ribosome biogenesis, EMG1, causes Bowen-Conradi syndrome Am J Hum Genet 2009 84 728 739 10.1016/j.ajhg.2009.04.017 19463982 PMC2694972

[B13] Ding H Schertzer M Wu X Gertsenstein M Selig S Kammori M Pourvali R Poon S Vulto I Chavez E Tam PP Nagy A Lansdorp PM Regulation of murine telomere length by Rtel: an essential gene encoding a helicase-like protein Cell 2004 117 873 886 10.1016/j.cell.2004.05.026 15210109

[B14] Palmieri SL Peter W Hess H Scholer HR Oct-4 transcription factor is differentially expressed in the mouse embryo during establishment of the first two extraembryonic cell lineages involved in implantation Dev Biol 1994 166 259 267 10.1006/dbio.1994.1312 7958450

[B15] Chambers I Colby D Robertson M Nichols J Lee S Tweedie S Smith A Functional expression cloning of Nanog, a pluripotency sustaining factor in embryonic stem cells Cell 2003 113 643 655 10.1016/S0092-8674(03)00392-1 12787505

[B16] Mitsui K Tokuzawa Y Itoh H Segawa K Murakami M Takahashi K Maruyama M Maeda M Yamanaka S The homeoprotein Nanog is required for maintenance of pluripotency in mouse epiblast and ES cells Cell 2003 113 631 642 10.1016/S0092-8674(03)00393-3 12787504

[B17] Beck F Erler T Russell A James R Expression of Cdx-2 in the mouse embryo and placenta: possible role in patterning of the extra-embryonic membranes Dev Dyn 1995 204 219 227 8573715 10.1002/aja.1002040302

[B18] Zhang J Tomasini AJ Mayer AN RBM19 is essential for preimplantation development in the mouse BMC Dev Biol 2008 8 115 10.1186/1471-213X-8-115 19087264 PMC2627835

[B19] Lerch-Gaggl A Haque J Li J Ning G Traktman P Duncan SA Pescadillo is essential for nucleolar assembly, ribosome biogenesis, and mammalian cell proliferation J Biol Chem 2002 277 45347 45355 10.1074/jbc.M208338200 12237316

[B20] Newton K Petfalski E Tollervey D Caceres JF Fibrillarin is essential for early development and required for accumulation of an intron-encoded small nucleolar RNA in the mouse Mol Cell Biol 2003 23 8519 8527 10.1128/MCB.23.23.8519-8527.2003 14612397 PMC262675

[B21] Chen H Li Z Haruna K Li Z Li Z Semba K Araki M Yamamura K Araki K Early pre-implantation lethality in mice carrying truncated mutation in the RNA polymerase 1-2 gene Biochem Biophys Res Commun 2008 365 636 642 10.1016/j.bbrc.2007.11.019 18023416

[B22] Adachi K Soeta-Saneyoshi C Sagara H Iwakura Y Crucial role of Bysl in mammalian preimplantation development as an integral factor for 40S ribosome biogenesis Mol Cell Biol 2007 27 2202 2214 10.1128/MCB.01908-06 17242206 PMC1820511

[B23] Romanova LG Anger M Zatsepina OV Schultz RM Implication of nucleolar protein SURF6 in ribosome biogenesis and preimplantation mouse development Biol Reprod 2006 75 690 696 10.1095/biolreprod.106.054072 16855206

[B24] Matsson H Davey EJ Draptchinskaia N Hamaguchi I Ooka A Leveen P Forsberg E Karisson S Dahl N Targeted disruption of the ribosomal protein S19 gene is lethal prior to implantation Mol Cell Biol 2004 24 4032 4037 10.1128/MCB.24.9.4032-4037.2004 15082795 PMC387766

[B25] Geuskens M Alexandre H Ultrastructural and autoradiographic studies of nucleolar development and rDNA transcription in preimplantation mouse embryos Cell Differ 1984 14 125 134 10.1016/0045-6039(84)90037-X 6467377

[B26] Flechon JE Kopecny V The nature of the 'nucleolus precursor body' in early preimplantation embryos: a review of fine-structure cytochemical, immunocytochemical and autoradiographic data related to nucleolar function Zygote 1998 6 183 191 10.1017/S0967199498000112 9770784

[B27] Zatsepina O Baly C Chebrout M Debey P The step-wise assembly of a functional nucleolus in preimplantation mouse embryos involves the cajal (coiled) body Dev Biol 2003 253 66 83 10.1006/dbio.2002.0865 12490198

[B28] Flechon JE Kopecny V The nature of the 'nucleolus precursor body' in early preimplantation embryos: a review of fine-structure cytochemical, immunocytochemical and autoradiographic data related to nucleolar function Zygote 1998 6 183 191 10.1017/S0967199498000112 9770784

[B29] Castle CD Cassimere EK Lee J Denicourt C Las1L Is a Nucleolar Protein Required for Cell Proliferation and Ribosome Biogenesis Mol Cell Biol 2010 30 4404 4414 10.1128/MCB.00358-10 20647540 PMC2937536

[B30] Panic L Montagne J Cokaric M Volarevic S S6-haploinsufficiency activates the p53 tumor suppressor Cell Cycle 2007 6 20 24 17245121 10.4161/cc.6.1.3666

[B31] Pestov DG Strezoska Z Lau LF Evidence of p53-dependent cross-talk between ribosome biogenesis and the cell cycle: effects of nucleolar protein Bop1 on G(1)/S transition Mol Cell Biol 2001 21 4246 4255 10.1128/MCB.21.13.4246-4255.2001 11390653 PMC87085

[B32] Rubbi CP Milner J Disruption of the nucleolus mediates stabilization of p53 in response to DNA damage and other stresses EMBO J 2003 22 6068 6077 10.1093/emboj/cdg579 14609953 PMC275437

[B33] Jones NC Lynn ML Gaudenz K Sakai D Aoto K Rey JP Glynn EF Ellington L Du C Dixon J Dixon MJ Trainor PA Prevention of the neurocristopathy Treacher Collins syndrome through inhibition of p53 function Nat Med 2008 14 125 133 10.1038/nm1725 18246078 PMC3093709

[B34] Barkic M Crnomarkovic S Grabusic K Bogetic I Panic L Tamarut S Cokaric M Jeric I Vidak S Volarevic S The p53 tumor suppressor causes congenital malformations in Rpl24-deficient mice and promotes their survival Mol Cell Biol 2009 29 2489 2504 10.1128/MCB.01588-08 19273598 PMC2682053

[B35] Ogushi S Palmieri C Fulka H Saitou M Miyano T Fulka J Jr The maternal nucleolus is essential for early embryonic development in mammals Science 2008 319 613 616 10.1126/science.1151276 18239124

[B36] Lowry RB Innes AM Bernier FP McLeod DR Greenberg CR Chudley AE Chodirker B Marles SL Crumley MJ Loredo-Osti JC Morgan K Fujiwara TM Bowen-Conradi syndrome: a clinical and genetic study Am J Med Genet 2003 120A 423 428 10.1002/ajmg.a.20059 12838567

[B37] Wu X Ding H Generation of conditional knockout alleles for PDGF-C Genesis 2007 45 653 657 10.1002/dvg.20339 17941048

[B38] Jacks T Remington L Williams BO Schmitt EM Halachmi S Bronson RT Weinberg RA Tumor spectrum analysis in p53-mutant mice Curr Biol 1994 4 1 7 10.1016/S0960-9822(00)00002-6 7922305

[B39] Ding H Wu X Kim I Tam PP Koh GY Nagy A The mouse Pdgfc gene: dynamic expression in embryonic tissues during organogenesis Mech Dev 2000 96 209 213 10.1016/S0925-4773(00)00425-1 10960785

[B40] Piette D Hendrickx M Willems E Kemp CR Leyns L An optimized procedure for whole-mount in situ hybridization on mouse embryos and embryoid bodies Nat Protoc 2008 3 1194 1201 10.1038/nprot.2008.103 18600225

[B41] Bassam BJ Gresshoff PM Silver staining DNA in polyacrylamide gels Nat Protoc 2007 2 2649 2654 10.1038/nprot.2007.330 18007600

[B42] Strezoska Z Pestov DG Lau LF Functional inactivation of the mouse nucleolar protein Bop1 inhibits multiple steps in pre-rRNA processing and blocks cell cycle progression J Biol Chem 2002 277 29617 29625 10.1074/jbc.M204381200 12048210

