# Supplementary figures and images for: EMG1 is essential for mouse pre-implantation embryo development
Source: BMC Dev Biol. 2010 Sep 21;10:99. doi: 10.1186/1471-213X-10-99 (PMC2954994; doi:10.1186/1471-213X-10-99)

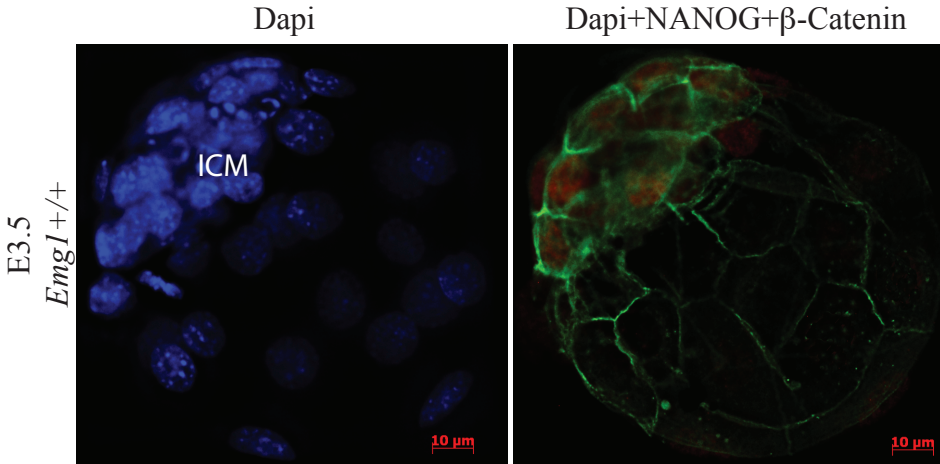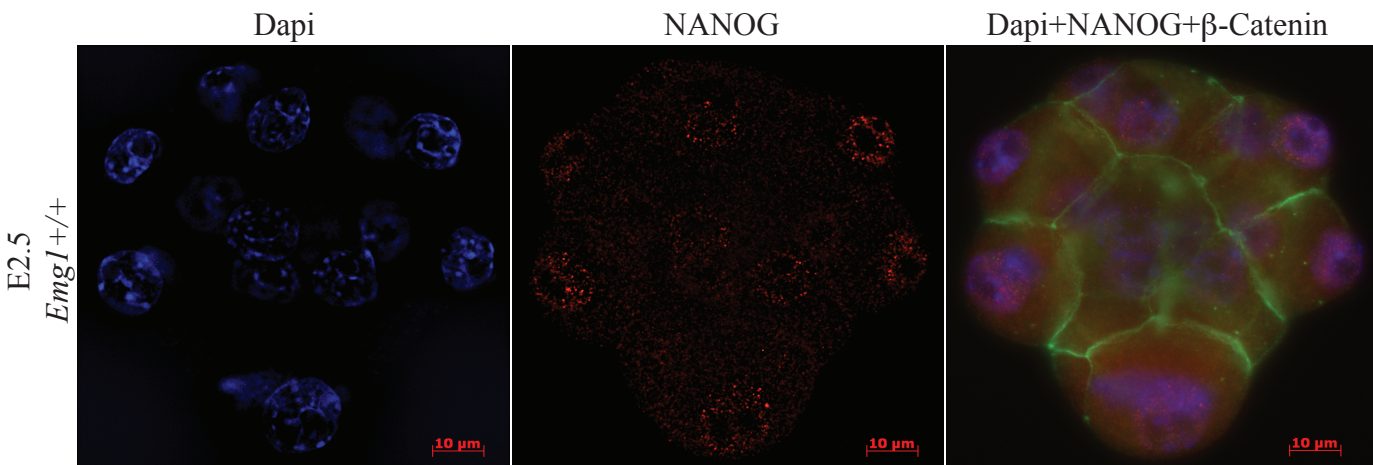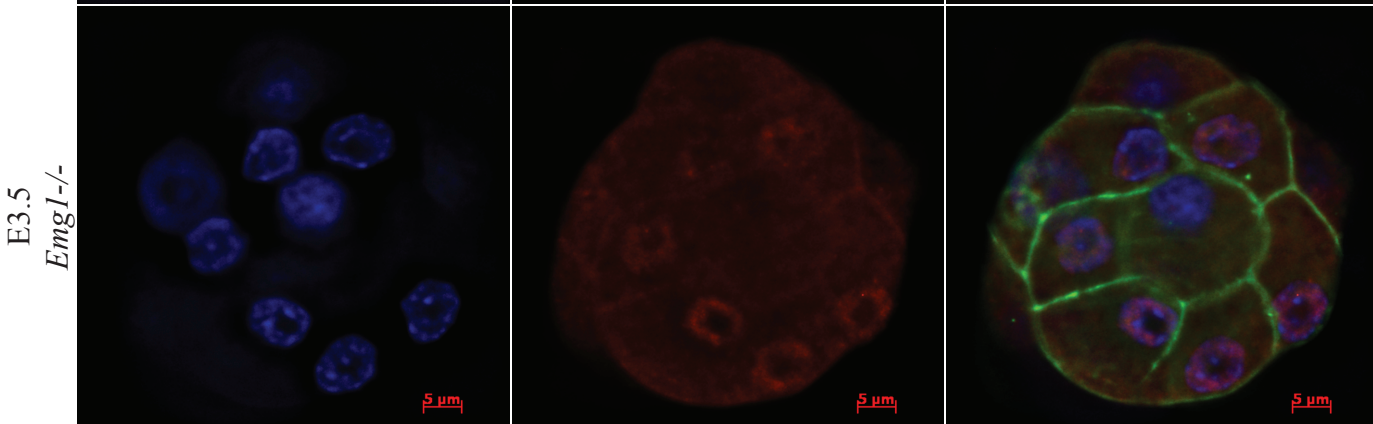

Supplement: Additional file 1 — Expression of NANOG in Emg1-/- mutant embryos. E3.5 embryos from Emg1+/- intercross were co-immunostained with anti-NANOG (red) and anti-β-catenin (green) antibodies. In E3.5 Emg1+/+ blastocysts, nuclear-localized NANOG is mainly found in the ICM. NANOG is also detected in the blastomeres of E2.5 Emg1+/+ morulae. At E3.5, Emg1-/- embryos arrest at the morula stage, in which the blastomeres express similar levels of NANOG as that in E2.5 Emg1+/+ morulae. [file 1471-213X-10-99-S1.PDF]
