# Supplementary material for: EMG1 is essential for mouse pre-implantation embryo development
Source: BMC Dev Biol. 2010 Sep 21;10:99. doi: 10.1186/1471-213X-10-99 (PMC2954994; doi:10.1186/1471-213X-10-99)
Supplement: Additional file 2 — p53 inactivation fails to rescue the pre-implantation arrest of the Emg1 null allele. E3.5 embryos were collected from intercross of Emg1+/-/p53+/- (A) and cross of Emg1+/-/p53-/-(male) with Emg1+/-/p53+/- (female) (B). In both, Emg1-/-/p53-/- embryos show the same morula arrest as Emg-/-/p53+/- or Emg-/-embryos. Scale bar, 100μm. [file 1471-213X-10-99-S2.PDF]

A B

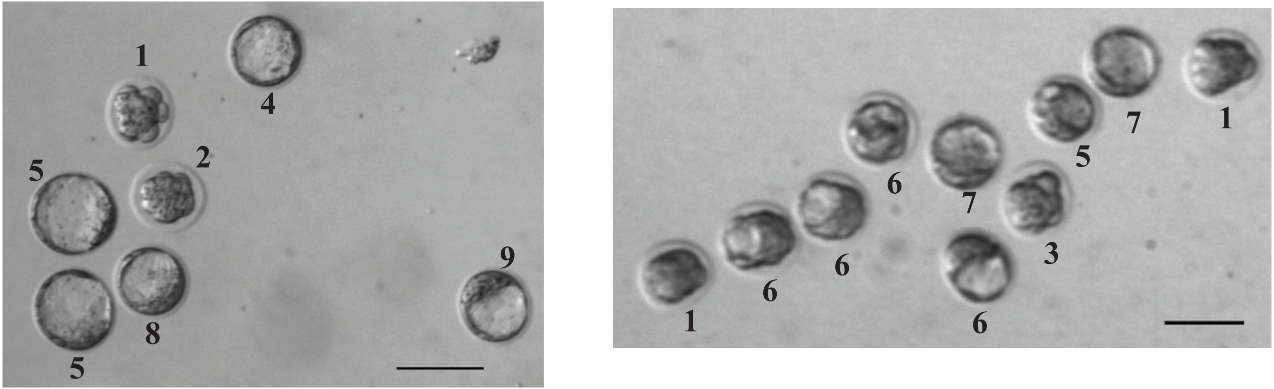

1: *Emg1-/-/p53-/-*
2: *Emg1-/-*
3: *Emg1-/-/p53+/-*
4: *p53+/-*
5: *p53-/-*
6: *Emg1+/-/p53+/-*
7: *Emg1+/-/p53-/-*
8: *Emg1+/-*
9: *wild-type*
